# Depression Symptoms Improve after Successful Weight Loss with Emotional Freedom Techniques

**DOI:** 10.1155/2013/573532

**Published:** 2013-07-28

**Authors:** Peta Stapleton, Dawson Church, Terri Sheldon, Brett Porter, Cassandra Carlopio

**Affiliations:** ^1^Department of Psychology, School of Humanities (Psychology), Bond University, Gold Coast, QLD 4229, Australia; ^2^Foundation for Epigenetic Medicine, Fulton, CA, USA; ^3^The Lakeside Rooms, Robina, QLD 4226, Australia; ^4^Mullumbimby Psychology, Mullumbimby, NSW 2482, Australia

## Abstract

Ninety-six overweight or obese adults were randomly allocated to a four-week EFT treatment or waitlist condition. Waitlist participants crossed over to the EFT group upon completion of wait period. Degree of food craving, perceived power of food, restraint capabilities, and psychological symptoms were assessed at pretreatment, posttreatment and at 12-month follow-up for combined EFT groups. Significant improvements in weight, body mass index, food cravings, subjective power of food, craving restraint and psychological coping for EFT participants from pretreatment to 12-month follow-up (*P* < 0.05) were reported. The current paper isolates the depression symptom levels of participants, as well as levels of eight other psychological conditions. Significant decreases from pre- to posttreatment were found for depression, interpersonal sensitivity, obsessive-compulsivity, paranoid ideation, and somatization (*P* < 0.05). Significant decreases from pretreatment to 12-month follow-up were found for depression, interpersonal sensitivity, psychoticism, and hostility. The results point to the role depression, and other mental health conditions may play in the successful maintenance of weight loss.

## 1. Introduction

A number of studies have noted an association between depression and weight loss. A study of 487 obese individuals found that weight loss was associated with a sustained reduction in depressive symptom levels, noting that obesity “causes or exacerbates depression” [1, page 2058]. A ten-year follow-up of bariatric surgery patients found sustained improvements in depression though not in anxiety [2]. Depression levels are a predictor of weight regain after dieting [3].

EFT (emotional freedom techniques) combines elements of cognitive therapy, exposure therapy, and acupuncture point stimulation [4]. Rather than using acupuncture needles, it relies on manual stimulation of the acupoints which is typical of Shiatsu or other forms of acupressure massage. Pressure on acupoints has been found to be as efficacious as acupuncture needling [5]. EFT uses a tapping technique (with two fingers) to stimulate pressure points on the face and upper body and a cognitive element which involves the person stating their present concern out loud as they perform the tapping [4]. It is widely understood that the parts of the brain involved in hyperarousal include the amygdala, and recent studies of the use of EFT have indicated a decrease in amygdala and hippocampus activity [4].

EFT has an extensive research bibliography (http://www.EFTuniverse.com/) that includes successful treatment of a variety of psychological conditions, including depression, anxiety, phobias, and PTSD. Treatment time frames are typically brief between one and ten sessions. Phobias are usually treated in a single session [6–8], while PTSD may require between four and ten sessions [9, 10].

EFT has been assessed for weight loss, weight maintenance, cravings, and addiction in a number of studies. A randomized controlled trial was conducted by the first author of this paper and found that there was a significant difference between pretreatment, posttreatment, and at 12-month follow-up in participants' weight, body mass index, food cravings, a person's subjective power over food, level of restraint, and overall psychopathology (*P* < 0.05) [11]. Results indicated that, for 96 participants, EFT demonstrated a significant change across these measures over time. 

In an outcome study, Church and Brooks examined addictive cravings for items like chocolate, food, alcohol, and tobacco in healthcare workers [12]. The 216 subjects participated in a day-long EFT group workshop at one of five professional conferences. The declines in cravings were both clinically and statistically significant (−83%, *P* < 0.0001). The same research team also measured psychological symptoms in a sample with self-identified craving and addiction problems attending a two-day group workshop which focused on these issues. They found improvements across a spectrum of mental health conditions, including depression and anxiety [13].

Internet-based research has been increasing over the course of the past decade and has now been used to examine EFT and weight loss. Church and Wilde examined depression and anxiety, as well as cravings and weight loss, in a sample (*N* = 72) enrolled in an online program called Skinny Genes [14]. The program uses EFT to address emotional eating in a six-week course. They compared psychological characteristics and weight before and after the program, as well as conducting a six-month follow-up. They found a decrease in depressive symptoms that was both clinically and statistically significant (−44%, *P* < 0.001). Skinny Genes participants lost an average of 12 lb during the six weeks of the program and a further 3 lb in the subsequent six months (*P* < 0.001).

EFT has been successfully utilized in the treatment of depression across a wide range of populations and delivery methods. A randomized controlled trial used EFT to address depressive symptoms in a population of teenage college students [15]. Assessment was performed using the Beck Depression Inventory (BDI) [16]. The sample (*N* = 18) scored in the “moderate to severe” range on intake, with the EFT group exhibiting a mean BDI score of 23.44. They then received four 90 min group EFT sessions over a period of three weeks. Afterwards, scores on the BDI were in the “nondepressed” range, with a mean of 6.08 (*P* = 0.001). 

 When depression was examined in a population of veterans whose PTSD symptoms were successfully remediated using EFT, their scores were in the high clinical range before treatment [17]. After six sessions of EFT, depression scores approached the “normal” range. When EFT was administered to a group of fibromyalgia patients via an online delivery method, depression levels declined significantly [18]. While the above are examples of randomized controlled trials, within-subjects outcome studies have yielded similar results for depression, as well as other psychological conditions [19, 20]. The study of 216 healthcare workers summarized above also examined depressive symptoms [12]. It found a clinically and statistically significant drop on posttest, with participant gains maintained on six-month follow-up (*P* < 0.0001).

A pilot study of veterans with comorbid PTSD and depression found a significant reduction in depression after 6 EFT sessions (*P* < 0.001) [21]. Another pilot study with veterans who received a week-long treatment intensive found posttest scores for depression significantly lower than pretest (*P* < 0.005) [10]. The study by Rowe found that a weekend group EFT seminar significantly reduced depression (*P* < 0.0001) [10]. In all these studies, participants maintained their improvements during follow-up periods of between three months and one year.

Stapleton et al. [11] used the SA-45 to assess psychological symptoms [22, 23]. This valid and reliable instrument contains two scales for the depth and breadth of psychological distress, the Global Symptom Index (GSI), and Positive Symptom Total (PST). It is a short form of the Symptom Checklist (SCL-90) and uses the proven items and structure of the SCL-90-R to create a brief measure of psychiatric symptomatology. The SA-45 uses a five-point Likert scale and measures anxiety, depression, hostility, interpersonal sensitivity, obsessive-compulsivity, paranoid ideation, phobic anxiety, psychoticism, and somatization [22, 23]. Significant improvements in both were found, both on posttest and on 6-month and 12-month follow-up. The current study isolates the previously unreported data measured by the nine subscales assessed by the SA-45: anxiety, depression, phobic anxiety, hostility, interpersonal sensitivity, psychoticism, obsessive-compulsivity, paranoid ideation, and somatisation. 

A number of studies have elucidated the physiological mechanisms of action of EFT. A randomized controlled trial examining the salivary cortisol levels of 83 subjects immediately before and 30 minutes after receiving a single session of either EFT, talk therapy, or rest found substantial reductions in cortisol levels, along with decreases in anxiety and depression [24]. EFT's use of acupressure points to reinforce its cognitive and exposure components is supported by trials showing reductions in fear in subjects receiving acupoint stimulation. When functional magnetic resonance imaging (fMRI) is used to measure the effect of acupuncture on the brain, a modulation of the stress response, by downregulating hyperarousal of the amygdala specifically and the limbic system generally, is found [25–27]. Fang et al. summarize their findings to state that acupuncture produces “extensive deactivation of the limbic-paralimbic-neocortical system” [26].

Several EFT studies have used electroencephalography (EEG) to demonstrate a downregulation of those brain frequencies associated with anxiety [28–30]. Feinstein summarized the evidence for EFT, hypothesizing “that (a) tapping on selected acupoints (b) during imaginal exposure (c) quickly and permanently reduces maladaptive fear responses to traumatic memories and related cues” [31]. A series of review papers has elucidated the neuronal, genetic, neurotransmitter, and hormonal pathways that may be engaged when EFT alleviates depression and other forms of psychological distress [31–33].

## 2. Methods

Ethics approval was obtained for the current study from the lead author's affiliated university at the time of the study (Griffith University). A total of 158 participants were screened for food craving severity and body mass index (BMI) status, and 38 were excluded for not meeting eligibility of being over the age of 18 years, with a BMI greater than 25, and not meeting any diagnosis of diabetes, hypoglycaemia, pregnancy, and taking psychotropic medication. A final group of 120 participants were offered a place, and 24 withdrew for personal reasons, leaving 96 to be randomized into the EFT treatment or waitlist condition. The waitlist condition waited without treatment for the length of the four-week trial and then completed the EFT treatment condition. All 96 participants completed an informed consent process. 

The Symptom Assessment 45 (SA-45) was used to assess symptomatology across nine psychiatric domains and is a short form of the Symptom Checklist (SCL-90) [34]. The SA-45 is a self-report questionnaire consisting of 45 questions that measures nine basic psychopathological dimensions using nine scales: anxiety, depression, hostility, interpersonal sensitivity, obsessive-compulsivity, paranoid ideation, phobic anxiety, psychoticism, and somatization. It consists of 45 items and is scored on a five-point Likert scale (0 = nothing, 1 = a little, 2 = moderate, 3 = quite a lot, and 4 = a lot). The SA-45 also features a Positive Symptom Index (PST) which indicates the total number of symptoms reported to be present (i.e., item yielding a response other than “Not at all”) and a Global Severity Index (GSI) which represents the total of the item response values for all items on the SA-45. It is a proven measure with sound psychometric properties and commonly used to assess clinical outcomes [35]. A self-report questionnaire package also included a demographic questionnaire, the Food Craving Inventory [36], the Power of Food Scale [37], and the SA-45. Three aspects of eating behaviour (cognitive restraint, uncontrolled eating, and emotional eating) were also measured using the Revised Restraint Scale [38]. The results of the food craving issues are reported elsewhere, but, in sum, significant improvements occurred in weight, body mass index, food cravings, subjective power of food, and craving restraint for EFT participants from pretreatment to 12-month follow-up [11, 39].

## 3. Treatment Condition

The EFT treatment was offered in groups of 15 participants and was conducted by a practitioner certified in EFT. The intervention consisted of four sessions (two hours duration each) with homework and was based on standardized treatment protocols [4, 40]. Full instructions and safeguards are described in Flint et al. [41]. The specific session topics were (1) psychoeducation about EFT and how it works; (2) the nature of food cravings and how they can be addressed with EFT; (3) feelings and food, and (4) relapse prevention, using EFT for stress and relaxation and goal setting. 

A treatment fidelity plan was formed prior to the trial commencing. The chief investigators who were also certified practitioners of EFT and the EFT practitioner delivering the treatment developed and reviewed the treatment manually to ensure that the active ingredients of the EFT intervention were fully operationalized according to Craig [4]. Fidelity to the manualized procedures was enforced and assessed by using intervention checklists, and participants completed social validity scales each week to evaluate the program and the EFT practitioner. On a seven-point Likert scale where 0 represented “not at all,” 91% of participants indicated EFT addressed their concerns “very well,” and 89% found the EFT program to “very much” meet their goals for reducing food cravings. On a five-point Likert scale, where 0 equaled “not at all,” 95% found the facilitator to be very approachable.

## 4. Data Analysis

Data was analyzed using SPSS-19 [42]. Individuals with missing data at any time point were excluded on an analysis-by-analysis basis. To test whether the effect of EFT on psychological distress symptoms was sustained at 12-month follow-up, repeated measure ANOVAs were used to measure the effect of three levels of the repeated measure variable of time (pretreatment, posttreatment, and at 12-month follow-up). The outcome variables were weight, BMI, SA-45 GSI score, SA-45 PST score, and all subscales of the SA-45 (anxiety, depression, hostility, interpersonal sensitivity, obsessive-compulsivity, paranoid ideation, phobic anxiety, psychoticism, and somatization). To maximize the sample size, the two groups EFT and WL were collapsed into one group (the WL received the active treatment after completion of the wait period). Paired comparisons between time-points were undertaken using post hoc tests. The Bonferroni correction was applied for multiple comparisons. 

Analysis was based on a total of 96 participants (EFT *n* = 49; WL *n* = 47). Twelve-month data was available for 43 participants (23 in EFT and 20 in WL groups); however, analyses were based on a lower number (39-40 total) if there was missing data for a specific variable (at pretreatment, posttreatment, or at 12-month follow-up). Reasons for attrition included move of residence and changes in electronic addresses (email) and telephone contact details, which prevented follow-up questionnaires being delivered. For all participants, contact was attempted at each point a total of three times. No adverse events were reported to any of the researchers by any participant.

## 5. Results

The majority of participants were female (89%), over 40 years of age (68%), married (53.1%), did not live alone (87%), had undertaken some form of education since high school (60%), were employed (79%), and had a household income exceeding $50,000 (65%). There were no significant differences between the EFT and WL conditions in baseline sociodemographic characteristics (*P* > 0.05). There were also no significant differences in the recruitment weight (mean 90.52 ± 19.11 kg or 199.1 ± 42 lbs), BMI (mean 32.78 ± 6.03), and self-report measures between the EFT and WL groups (*P* > 0.05).

Table 1 shows the means, standard deviations at pretreatment, posttreatment, and at 12-month follow-up for the collapsed groups. The premean scores for the SA-45 subscales of depression, hostility, interpersonal sensitivity, and psychoticism all had symptom domain scale *T*-scores less than 65, indicating a low base-rate group without identified behavioral problems. However, in clinical contexts, *T*-scores over 60 are likely to indicate the presence of significant problem areas. Because overweight and obesity are typically linked to a range of physical and psychological concerns [39], this *T*-score cut-off is worth noting.

There was a significant difference between pretreatment, posttreatment, and at 12-month follow-up across participants' weight, BMI, SA-45 GSI and SA-45 PST scores, and the subscales scores for depression, hostility, and interpersonal sensitivity (*P* < 0.05) indicating participants demonstrated significant change over time. In order to see where the differences were occurring, post hoc paired comparisons, with Bonferroni adjustment, for repeated measures ANOVAs were conducted (see Table 2). There were significant decreases from pre- to posttreatment for the SA-45 GSI score only (mean difference −2.96, *P* < 0.05). There were significant reductions from pretreatment to 12-month follow-up for participants' weight (mean difference −5.05 kg or 11.1 lbs, *P* < 0.05), BMI (mean difference −2.28, *P* < 0.05), SA-45 GSI (mean difference −5.47, *P* < 0.05), depression (mean difference 6.38, *P* < 0.05), and interpersonal sensitivity scores (mean difference 4.84, *P* < 0.05). From posttreatment versus 12-month follow-up, there were significant reductions for participants' weight (mean difference −4.65 kilograms/10.2 pounds, *P* < 0.05) and BMI (mean difference −2.13, *P* < 0.05). There were no significant differences for the SA-45 GSI and SA-45 PST scores or the subscales from posttreatment to 12-month follow-up.

In summary, the EFT treatment resulted in a statistically significant reduction of the SA-45 GSI score which is a descriptor of overall psychopathology, in participants over the 4-week treatment program. It can also be concluded that over the 12-month period beyond treatment, reductions in weight, BMI, and GSI scores were statistically significantly lower than at the pretest phase. The depression scores for participants over 12 months improved significantly, as did participants' symptomatic feelings about themselves in relation to others (interpersonal sensitivity).

## 6. Discussion

The relationship between depression and weight has been the subject of an extensive body of research, pertinent findings of which are noted in the introduction to this paper. They demonstrate the frequent comorbidity of depression and obesity. An intervention that reduces depressive symptoms can support the treatment of obesity.

The literature demonstrates a weaker statistical link between anxiety and obesity than is the case for depression, though anxiety nevertheless seems to play an important clinical role. The current study reinforces these distinctions, finding a robust statistical link between depression in a weight loss program (*P* < 0.036) but not for anxiety.

The clinical importance of this study is the demonstration that depressive symptoms improve in a group that is simultaneously losing weight. Some of the studies reviewed in the introductory literature attempt to parse the question of whether weight loss precedes improvement in depression or vice versa. The current study suggests that both goals can be pursued simultaneously and that both may mutually reinforce the other. From the point of view of the client, weight loss may remove a source of depressive ideation, while reduced depression may indicate emotional changes which reduce cravings and unrestrained eating.

Research has shown a link between levels of the stress hormone cortisol and both depression and obesity. Elevated cortisol levels are linked to the accumulation of centrally distributed adipose tissue [43]. Depression is also associated with elevated cortisol [44]. These reviews include studies demonstrating a wide variety of cooccurring adverse health effects, such as metabolic dysregulation, elevated blood pressure, cholesterol, and insulin.

Church et al. found a significant reduction in salivary cortisol levels after an hour-long EFT session [24]. It might therefore be possible that one mechanism by which EFT improves both depression and obesity is the reduction of cortisol. Further research might incorporate biological measures of EFT's efficacy, such as cortisol, EEG, and heart rate variability. 

Weight regain after weight loss is an often-reported problem, and there is a general public perception that weight regain is inevitable. Research shows, however, that a percentage of those who lose weight keep it off successfully [45]. A database maintained by the National Weight Control Registry shows that 20% of individuals who lose 10% or more of their body weight keep it off for a year or more [1].

One feature of previous studies of EFT for weight loss is the decline in weight during the follow-up period. Stapleton et al. [11] found that, in the year following the weight loss program, participants lost an additional 11 lb. Church and Wilde [14] found that participants lost an additional 3 lb in the six months following the Skinny Genes online program. Skinny Genes does not focus on weight loss per se or prescribe any particular diet but instead focuses on the emotional aspects of eating. The authors hypothesize that once an individual's emotional responses to food are modified, weight loss is a sequel. These studies point to the contribution made by EFT in reducing depression. Wing and Phelan [1] noted an association between reduced depression and successful long-term weight loss. McGuire et al. [3] found that reduced depression was a reliable predictor of weight loss maintenance.

The limitations of this study include the lack of an evidence-based active control condition, such as cognitive behavior therapy. Other limitations include the possibility that the results were exaggerated by expectancy effects, therapist allegiance to the EFT method, a placebo effect, and a modest sample size. Physical activity was not assessed or measured in the present study and thus may have been a confounding variable. The generalizability of the results to men is further limited by the small number of male participants (*n* = 10). Despite these limitations, the study makes a contribution to the understanding of the association between weight loss and depression and the efficacy of a brief and cost-effective self-help technique to the long-term maintenance of weight loss.

As a relatively new and innovative treatment approach, EFT is gathering evidence as a sound and efficacious intervention for a range of clinical and health problems. Empirical support now exists for this self-administered approach, and given its similarities to other protocols which aim to extinguish a stress response by pairing it with an incompatible response (e.g., systematic desensitization), EFT as a brief psychological exposure technique may become an additional tool for treating a wide variety of concerns.

## Figures and Tables

**Table 1:** Means, standard deviations at pretreatment, posttreatment, and at 12-month follow-up for collapsed groups.

	*N*	Pretreatment		Posttreatment		12-month follow-up		df	F	P
		Mean	SD	Mean	SD	Mean	SD			
Weight*	40	92.25 kg/203 lbs	18.73	91.85 kg/202.1 lbs	18.88	87.20 kg/191.8 lbs	18.28	[1.04, 40.70]	9.81	0.003*
BMI*	40	33.00	6.60	32.85	6.83	30.73	6.56	[1.04, 40.57]	13.73	0.001*
SA-45GSI*	45	59.20	9.7	56.24	7.7	53.73	9.6	[2, 88]	8.93	<0.001*
SA-45PST*	45	58.56	9.54	56.09	8.34	53.93	10.52	[2, 88]	5.88	0.004*
SA-45 ANX	19	59.26	10.01	56.57	9.17	56.31	7.18	[2, 36]	1.46	0.24
SA-45 DEP*	19	61.42	7.88	58.57	7.42	55.05	6.72	[2, 36]	8.21	0.001*
SA-45 HOS*	19	60.21	6.62	58.89	6.34	56.73	4.12	[2, 36]	3.91	0.02*
SA-45 INT*	19	60.00	6.78	56.57	6.88	55.15	7.05	[2, 36]	5.46	0.008*
SA-45 OC	19	59.31	9.55	55.36	9.32	55.73	9.61	[2, 36]	2.93	0.066
SA-45 PAR	19	58.21	8.48	55.36	8.34	54.68	6.71	[2, 36]	2.31	0.114
SA-45 PHO	19	59.89	4.04	59.26	2.99	60.05	3.01	[2, 36]	0.66	0.56
SA-45 PSY	19	61.89	4.51	61.63	4.07	61.31	3.81	[2, 36]	0.28	0.75
SA-45 SOM	19	58.10	7.10	54.78	7.27	58.05	8.92	[2, 36]	1.58	0.22

*P < .0.05.

BMI: Body Mass Index.

SA-45GSI: Symptom Assessment Global Severity Index;

SA-45PST: Symptom Assessment Positive Symptom Index;

SA-45 ANX: Symptom Assessment Anxiety;

SA-45 DEP: Symptom Assessment Depression;

SA-45 HOS: Symptom Assessment Hostility;

SA-45 INT: Symptom Assessment Interpersonal Sensitivity;

SA-45 OC: Symptom Assessment Obsessive Compulsive;

SA-45 PAR: Symptom Assessment Paranoid Ideation;

SA-45 PHO: Symptom Assessment Phobic Anxiety;

SA-45 PSY: Symptom Assessment Psychoticism;

SA-45 SOM: Symptom Assessment Somatization.

**Table 2:** Post hoc paired comparisons, with Bonferroni adjustment, for repeated measures ANOVAs.

	*N*	12-month follow-up versus pretreatment		12-month follow-up treatment versus posttreatment		Post- versus pretreatment	
		Mean difference	P	Mean difference	P	Mean difference	P
Weight	40	−5.05 kg/11.1 lbs	0.006*	−4.65 kg/10.2 lbs	0.014*	−0.40 kg/0.9 lbs	0.407
BMI	40	−2.28	0.001*	−2.13	0.003*	−0.15	0.406
SA-45GSI	45	−5.47	0.001*	−2.51	0.257	−2.96	0.029*
SA-45PST	45	−4.62	0.013*	−2.16	0.334	−2.47	0.117
SA-45 ANX	19	2.94	0.46	−0.26	0.47	−2.68	0.47
SA-45 DEP	19	6.38	0.001*	−3.52	0.15	−2.84	0.28
SA-45 HOS	19	3.47	0.058	−2.15	0.41	−1.31	0.61
SA-45 INT	19	4.84	0.016*	−1.42	1.00	−3.42	0.10
SA-45 OC	19	3.57	0.25	0.36	1.00	−3.94	0.06
SA-45 PAR	19	3.56	0.19	−0.68	1.00	−2.84	0.13
SA-45 PHO	19	−0.15	1.00	0.78	0.59	−0.63	1.00
SA-45 PSY	19	0.56	1.00	−0.31	1.00	−0.26	1.00
SA-45 SOM	19	0.05	1.00	3.26	0.48	−3.31	0.16

*P < .0.05.

BMI: Body Mass Index;

SA-45GSI: Symptom Assessment Global Severity Index;

SA-45PST: Symptom Assessment Positive Symptom Index;

SA-45 ANX: Symptom Assessment Anxiety;

SA-45 DEP: Symptom Assessment Depression;

SA-45 HOS: Symptom Assessment Hostility;

SA-45 INT: Symptom Assessment Interpersonal Sensitivity;

SA-45 OC: Symptom Assessment Obsessive Compulsive;

SA-45 PAR: Symptom Assessment Paranoid Ideation;

SA-45 PHO: Symptom Assessment Phobic Anxiety;

SA-45 PSY: Symptom Assessment Psychoticism;

SA-45 SOM: Symptom Assessment Somatization.
